# Raman scattering yields cubic crystal grain orientation

**DOI:** 10.1038/s41598-019-45782-z

**Published:** 2019-06-28

**Authors:** K. Tesar, I. Gregora, P. Beresova, P. Vanek, P. Ondrejkovic, J. Hlinka

**Affiliations:** 1 Institute of Physics of the Czech Academy of Sciences, Na Slovance 2, Prague 8, 182 21 Czech Republic; 2 Czech Technical University in Prague, Faculty of Nuclear Sciences and Physical Engineering, Department of Materials, Trojanova 13, Praha 2, 120 00 Czech Republic

**Keywords:** Ceramics, Ferroelectrics and multiferroics, Phase transitions and critical phenomena, Structure of solids and liquids

## Abstract

The paper proposes a fully optical method for determination of a cubic crystal grain orientation in a sample inspected by a Raman microscope. The method is based on a universal and strong polarisation anisotropy of the Raman scattering by doubly degenerate optic phonon modes and it only requires a standard Raman microscope equipped with a polarisation analysis. Explicit formulas for the orientation of the crystal grain are derived. The feasibility of the approach is demonstrated by comparing grain orientations in a polycrystalline cubic lacunar spinel GaV_4_S_8_ determined independently using electron backscatter diffraction and Raman scattering methods.

## Introduction

Optical microscope is a standard laboratory tool for inspection of the sample geometry and its internal microstructure at *μ*m to mm dimensions. Raman microscope is an instrument that allows, on the top of it, recording Raman spectra from the area selected by the focus of the objective. In this way, Raman microscopy provides a complementary, spatially resolved information about the structure and chemical composition.

Another important characteristic of the investigated material could be the orientation of the crystal structure within the optically inspected area. For example, optically uniaxial domains in crystals exist in several orientation variants that can be easily distinguished by polarisation sensitivity of Raman scattering. Also the crystal grain orientations in optically uniaxial polycrystalline materials can be determined by Raman scattering. In particular, the angle between the local principal optical axis and the sample surface normal can be calculated from the fine spectral shifts caused by dipole-dipole interaction among polar Raman modes (oblique phonon mode method)^1^. Among others, combination of the oblique phonon mode method with the piezoforce scanning microscopy allowed us to assign the ferroelectric domain wall types in BiFeO_3_ ceramics^2^.

Let us stress that none of the above methods can be applied to cubic crystalline materials, because the optical indicatrix of cubic crystals does not have any special direction and these crystals have no oblique phonon eigenmodes. Fortunately, there is still another possibility, which is applicable to cubic crystals. We are suggesting here a method for detection of a completely arbitrary crystallite orientation by relying only on the universal polarisation dependence of Raman scattering by their doubly degenerate nonpolar phonon modes. The procedure is demonstrated here on a polycrystalline ceramic pellet of a cubic lacunar spinel GaV_4_S_8_. We have chosen GaV_4_S_8_ since it is a cubic substance with well-separated Raman active doublet modes and at the same time it is known to be one of the very few hosts of magnetoelectric skyrmion phases^3–5^. Currently, this fascinating lacunar spinel family is attracting more and more attention^6–11^. Nevertheless, the same approach is applicable for any other cubic material with a well-defined doubly degenerate Raman active optic phonon mode.

The paper is organised as follows. At first, we introduce GaV_4_S_8_ and summarise the known polarisation dependence of its Raman active modes. Then we briefly describe the orientation texture of our specimen obtained by the well established electron backscatter diffraction (EBSD) and we explain how the orientation can be obtained by Raman scattering only. Finally, we compare the results and conclude the paper by discussing the advantages of this fully optical method.

## Results

### Raman scattering spectra of GaV_4_S_8_

Our demonstration material GaV_4_S_8_ crystallizes in a non-centrosymmetric cubic structure with the $F\bar{4}3m\,({T}_{d}^{2})$ symmetry. Below about ≈40 K it undergoes a phase transition to a rhombohedral $R3m\,({C}_{3v}^{5})$ phase. This rhombohedral distortion, driven by Jahn-Teller effect in vanadium tetrahedra, causes ferroelectric polarisation in the low-temprature phase^3,4^. On the top of it, magnetic ordering with helicoidal and skyrmion^12,13^ arrangements observed at moderate magnetic fields and at temperatures below about 15 K attracted a great attention to the whole lacunar spinel family^3,4^.

**Figure 1:**
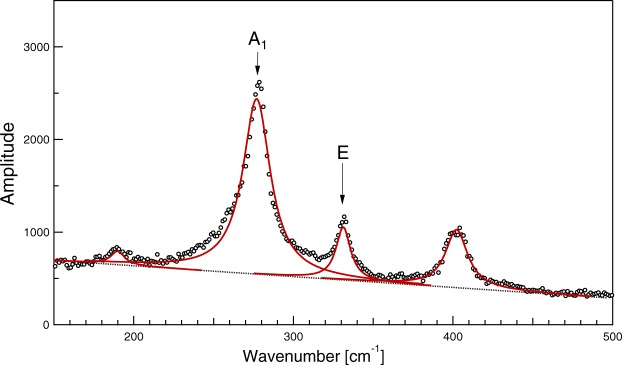
Typical parallel-polarised Raman spectrum detected from an arbitrarily oriented grain.

Here we only deal with the ambient temperature $F\bar{4}3m$ phase. The factor group analysis predicts 3A_1_ + 3E + 3F_1_ + 6F_2_ Brillouin zone centre optic modes there, out of which 3A_1_ + 3E are nonpolar Raman active modes and 6F_2_ are simultaneously Raman and IR active modes. Previous single crystal spectroscopic and theory investigations^14^ allowed us to estimate frequencies of all its Raman active modes. For the purpose of the grain orientation analysis, it is practical to inspect well separated and strongly scattering modes, such as the *A*_1_ phonon mode near 277 cm^−1^ and the *E* phonon mode near 330 cm^−1^ (see Fig. 1).

The modes belonging to different irreducible representations are known to be conveniently distinguishable by the polarisation analysis^15^. In the case of GaV_4_S_8_, within its natural crystallographic reference frame, Raman tensor *R* of the *A*_1_ modes has a general form of

1 $$R=(\begin{array}{ccc}a & 0 & 0\\ 0 & a & 0\\ 0 & 0 & a\end{array}).$$

Raman tensors *R*_*α*_ of the *E*-symmetry mode components read

2 $${R}_{1}=(\begin{array}{ccc}b & 0 & 0\\ 0 & b & 0\\ 0 & 0 & -2b\end{array}),\,{R}_{2}=(\begin{array}{ccc}-\sqrt{3}b & 0 & 0\\ 0 & \sqrt{3}b & 0\\ 0 & 0 & 0\end{array}),$$

and the Raman tensor of the *F*_2_ mode components can be expressed as

3 $${R}_{1}=(\begin{array}{ccc}0 & 0 & 0\\ 0 & 0 & c\\ 0 & c & 0\end{array}),\,{R}_{2}=(\begin{array}{ccc}0 & 0 & c\\ 0 & 0 & 0\\ c & 0 & 0\end{array}),\,{R}_{3}=(\begin{array}{ccc}0 & c & 0\\ c & 0 & 0\\ 0 & 0 & 0\end{array}).$$

In the following it will be important that the polarisation dependence of the first-order Raman scattering intensity by a phonon mode spanned by one or more orthogonal components *α* is given by a simple prefactor ${{\rm{\Lambda }}}_{{\rm{in}},{\rm{out}}}$ given by the sum of the squared moduli of the corresponding Raman tensors ${({R}_{\alpha })}_{ij}$, contracted by the incoming and outgoing photon polarisation vectors **e**_*in*_, **e**_*out*_, as follows

4 $${{\rm{\Lambda }}}_{{\rm{in}},{\rm{out}}}=\sum _{\alpha }\,|{{\bf{e}}}_{{\rm{in}}}{R}_{\alpha }{{\bf{e}}}_{{\rm{out}}}{|}^{2}.$$

In this paper, we have only considered parallel-polarised scattering configuration, where ${{\bf{e}}}_{in}={{\bf{e}}}_{out}={\bf{e}}$, and the equation (4) reduces to

5 $${\rm{\Lambda }}=\sum _{\alpha }\,|{\bf{e}}{R}_{\alpha }{\bf{e}}{|}^{2}.$$

**Figure 2:**
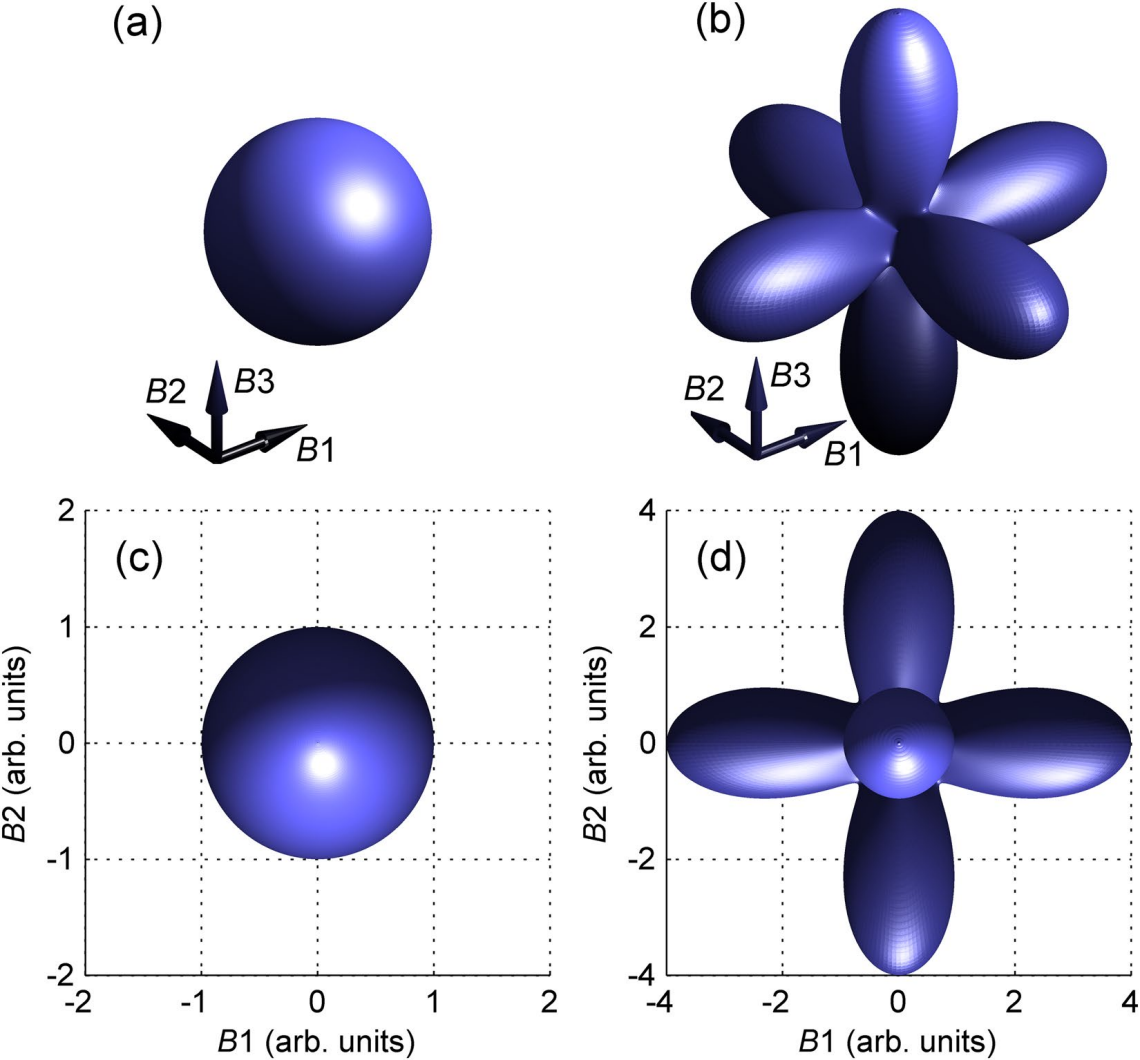
Polar plot of the polarisation-dependent factor ${\rm{\Lambda }}$ of Raman scattering by (**a**) *A*_1_ and (**b**) *E*-symmetry modes in a cubic crystal calculated using equations (1, 2, 4 and 5) for *a* = *b* = 1. *B*1 − *B*3 stand for the three perpendicular directions parallel to the fourfold symmetry axes of the crystal. Projections along the *B*3 axis are shown in (c and d) panels, respectively.

The polar plot of the parallel-polarised Raman intensity prefactor ${\rm{\Lambda }}$ is plot in Fig. 2 for the case of *A*_1_ and *E* modes, respectively. The scattering by the *E* mode is strongly enhanced when the photon polarisation is parallel to one of the fourfold axes. It is this universal strong anisotropy that is exploited in this paper in order to determine the orientation of the crystallographic axes within the scattering volume.

### Crystal grain orientations from EBSD

**Figure 3:**
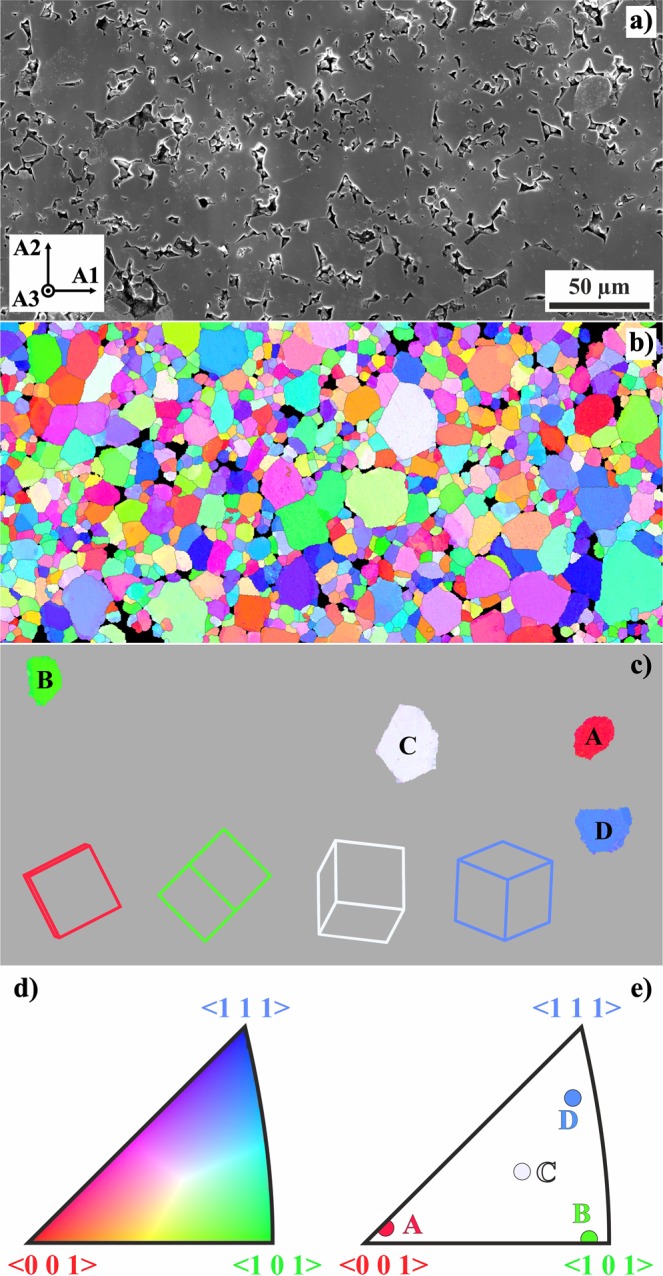
Grain morphology of the investigated GaV_4_S_8_ ceramics. (**a**) Scanning electron microscope image of an area of interest with sample coordinate system as an inset; (**b**) Inverse pole figure map from EBSD analysis of the area in (**a**); (**c**) positions of four grains chosen for detailed Raman measurements ($A:\{1\,\bar{15}\,1\}\langle \bar{5}\,\bar{1}\,\bar{10}\rangle$, $B:\{\bar{7}\,0\,8\}\langle 8\,\bar{11}\,7\rangle$, $C:\{7\,\bar{3}\,12\}\langle 0\,4\,1\rangle$, $D:\{\bar{17}\,14\,\bar{10}\}\langle 8\,9\,\bar{1}\rangle$), indicated projections of elementary cubic cells clarify their crystallographic orientation; (**d**) unit triangle with the color code of the inverse pole figure map; (**e**) surface normals of the chosen grains as determined from EBSD.

To inspect the GaV_4_S_8_ ceramics surface we used the EBSD technique. The scanning electron microscope image in Fig. 3a shows the surface morphology of the sample covering 250 × 500 *μ*m^2^. As can be seen, some smaller grains were removed from the surface by the polishing. Figure 3b shows an EBSD micrograph (inverse pole figure map) of the above area. The average grain size of this material is (6.8 ± 3.8) *μ*m, according to the EBSD data. However, it is necessary to point out that several grains within the map reach the size of ≈25 *μ*m. Some of these grains were thereafter chosen for further analysis. The selected grains, labelled A, B, C, D, are shown in Fig. 3c with their respective orientation. The colour coding of the inverse pole figure map refers to the standard unit triangle shown in Fig. 3d. The positions of grains A, B, C, D in this unit triangle are presented in Fig. 3e. The EBSD imaged area has been marked by focused ion beam (FIB) in order to facilitate its localisation under the optical microscope (not shown in Fig. 3).

In general, the crystal grain orientation relates the laboratory Cartesian reference frame *A*1 − *A*2 − *A*3 (see Fig. 3a) with the local Cartesian reference frame *B*1 − *B*2 − *B*3, attached to the crystal grain axes. The crystallographic orientation of the individual crystal grains identified by EBSD is typically given in the standard texture notation (*hkl*) [uvw].

The first three indices (*hkl*) define the crystallographic orientation of the sample surface facet (*A*3 in Fig. 3a) in the *B*1 − *B*2 − *B*3 frame. This local *B*1 − *B*2 − *B*3 Cartesian frame is attached to the three mutually perpendicular fourfold axes of the grain. Since the EBSD scattering intensity is invariant with respect to symmetry operations of the $m\bar{3}m$ Laue symmetry class of the $\bar{4}3m$ point group symmetry of the grain, one can choose the crystal grain axes *B*1 − *B*2 − *B*3 in a way that the Miller indices of the surface comply with

6 $$0\le k\le h\le l.$$

In this case, the local crystal grain coordinates of the surface unit normal **n** can be expressed as

7 $${\bf{n}}=({n}_{1},{n}_{2},{n}_{3})=\frac{(h,k,l)}{\sqrt{{h}^{2}+{k}^{2}+{l}^{2}}},$$

where the directional cosines are ordered and non-negative:

8 $$0\le {n}_{2}\le {n}_{1}\le {n}_{3}\le 1,\,1/\sqrt{3}\le {n}_{3}\le 1.$$

Each such vector **n** is then uniquely associated with one point in the inverse pole figure triangle (Fig. 3d).

The other part of the (*hkl*) [*uvw*] symbol defines the three Miller indices [*uvw*] of the conventionally chosen reference direction in the sample surface (*A*1 in the case of Fig. 3a). Local crystallographic coordinates of the corresponding unit vector **b** read

9 $${\bf{b}}=\frac{(u,v,w)}{\sqrt{{u}^{2}+{v}^{2}+{w}^{2}}}.$$

### Crystal grain orientations from Raman scattering

We have selected grains with larger top surfaces which have been well observable in the optical microscope (such as those marked in Fig. 3c). The Raman scattering intensity *I*, detected at the resonant frequency of the selected phonon mode, is mostly given by the first order Raman scattering by that mode. Therefore, its dependence on **e** is proportional to the corresponding factor ${\rm{\Lambda }}$ of equation (5). The angular dependence of ${\rm{\Lambda }}$, shown in Fig. 2, suggests that for an *E* symmetry mode the intensity *I* is maximised when **e** is close to the direction of one the local fourfold symmetry axes.

By rotation of the sample stage around the axis of the objective of the microscope, we could collect Raman spectra from the selected grain as a function of the oriented precession angle $\phi$ between the reference direction **b** and the photon polarisation ${\bf{e}}(\phi )$, where

10 $${\bf{e}}(\phi )={\bf{b}}\,\cos \,\phi +{\bf{t}}\,\sin \,\phi ,$$

where **t** = **n** × **b**.

**Figure 4:**
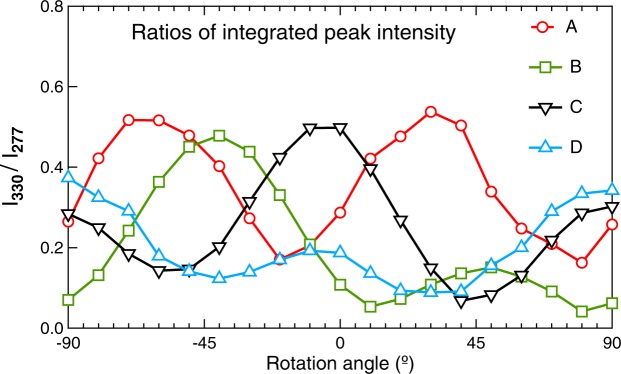
Parallel-polarised Raman scattering intensity ratios detected from the grain A (circles), B (squares) and C (triangles with vertex down) and D (triangles with vertex up) at selected frequencies as a function of the angle $\phi$ between the polariser and the reference direction *A*1 on the sample surface (indicated in Fig. 3). The intensity ratios are obtained from the raw measured data after the flat background subtraction as the integrated intensity at 330 ± 5 cm^−1^, divided by the integrated intensity at 277 ± 5 cm^−1^, *I*_330_/*I*_277_.

Since the scattering intensity at frequencies corresponding to the *A*_1_ is isotropic, it is convenient to get rid of overall geometrical factors by inspecting the *E* to *A*_1_ intensity ratio instead of the *E*-mode intensity alone. The angular dependence of such ratio *I*_330_/*I*_277_ of Raman scattering intensities at frequencies 330 and 277 cm^−1^, corresponding to the selected strong *A*_1_ and *E* modes, is shown in Fig. 4 for several selected grains. As expected, the angular dependence of the intensity near the frequency of the *E* mode is rather pronounced, and it allows us to extract the angles ${\phi }_{{\rm{m}}}$ and ${\phi }_{{\rm{m}}}$ at which the ${I}_{330}(\phi )$ has the highest and the second highest maxima.

For the sake of quantitative estimation of the crystal grain orientation, it is convenient to extract not only the angles ${\phi }_{{\rm{M}}}$ and ${\phi }_{{\rm{m}}}$ but also the corresponding values of ${I}_{{\rm{m}}}=I({\phi }_{{\rm{m}}})$ and ${I}_{{\rm{m}}}=I({\phi }_{{\rm{m}}})$. By comparing these values to the *E*-mode intensity *I*_0_ detected for a special configuration with ${\bf{e}}\parallel \langle 100\rangle$, one can derive directly the desired values of the reduced polarisation factor *λ*

11 $$\begin{array}{l}{\lambda }_{{\rm{M}}}=\lambda ({\phi }_{{\rm{M}}})={I}_{{\rm{M}}}/{I}_{0},\\ {\lambda }_{{\rm{m}}}=\lambda ({\phi }_{{\rm{M}}})={I}_{{\rm{m}}}/{I}_{0}.\end{array}$$

To achieve a higher precision, the spectra at ${\phi }_{{\rm{M}}}$ and ${\phi }_{{\rm{m}}}$ were fitted to the standard model of a superposition of damped harmonic oscillators response functions and the ratio of damped harmonic oscillator amplitude prefactors associated with the selected *E* to *A*_1_ modes was then used as the $I({\phi }_{{\rm{m}}})$ and $I({\phi }_{{\rm{m}}})$ values. The normalisation factor *I*_0_ has been determined here as the global maximum of *E* to *A*_1_ mode intensity ratios in the ensemble of all spectra taken in different orientations, but it could have been equally well set up from a spectrum measured on a selected calibration grain or on an oriented single crystal with a precisely known surface orientation.

In fact, each of the six lobes of the ${\rm{\Lambda }}$ function for an *E*-symmetry mode has an almost perfect rotational symmetry so that in the vicinity of the given fourfold axis it can be well approximated by an expression depending only on the deviation angle *δ* between **e** and that axis. After inserting the equation (2) into equation (5), it is convenient to express the unit polarisation vector **e** in spherical coordinates ${\bf{e}}=(\cos \,\delta \,\sin \,\varepsilon ,\,\sin \,\delta \,\sin \,\varepsilon ,\,\cos \,\varepsilon )$ of the local Cartesian reference frame *B*1 − *B*2 − *B*3 and by keeping only the leading-order terms in *δ* up to *o*(*δ*^2^), the dependence on the azimuthal angle $\varepsilon$ drops out and the reduced prefactor $\lambda ={\rm{\Lambda }}$/$4|b{|}^{2}$ can be expressed as

12 $$\lambda \doteq {|(\frac{1}{4}+\frac{3}{4}\cos 2\delta )|}^{2}.$$

Therefore, when the photon polarisation **e** is close to the direction of a fourfold axis, the corresponding angle *δ* can be determined from the reduced scattering intensity *λ* using the inverse of the equation (12),

13 $$\delta \doteq \frac{1}{2}\,\arccos \,[\frac{4}{3}\sqrt{\lambda }-\frac{1}{3}].$$

Since *δ*(*λ*) is also a monotonous function, it is clear that $\delta (\phi )$ has a minimum where $I(\phi )$ has a maximum, and, therefore, ${\delta }_{{\rm{m}}}=\delta ({\lambda }_{{\rm{m}}})$ and ${\delta }_{{\rm{m}}}=\delta ({\lambda }_{{\rm{m}}})$ correspond to the deviation of the closest and second closest local fourfold axes from the surface of the sample. In other terms, ${n}_{1}=\,\sin \,{\delta }_{{\rm{m}}}$ and ${n}_{2}=\,\sin \,{\delta }_{{\rm{M}}}$ and both angles in the arguments are taken positive due to the inequalities assumed in equation (8). The normalisation condition $|n|=1$ then allows us to determine the missing component *n*_3_. The surface normal in the local crystallographic basis of the *B*1 − *B*2 − *B*3 Cartesian frame can be thus expressed as

14 $${{\bf{n}}}_{{\rm{Raman}}}\doteq (\sin \,{\delta }_{{\rm{m}}},\,\sin \,{\delta }_{{\rm{M}}},\sqrt{1-{(\sin {\delta }_{{\rm{M}}})}^{2}-{(\sin {\delta }_{{\rm{m}}})}^{2}}).$$

Similarly, first two directional cosines of the unit vector *b* can be determined from the set of equations

15 $$\begin{array}{l}{\bf{e}}({\phi }_{{\rm{m}}})={\bf{b}}\,\cos \,{\phi }_{{\rm{m}}}+{\bf{t}}\,\sin \,{\phi }_{{\rm{m}}},\\ {\bf{e}}({\phi }_{{\rm{M}}})={\bf{b}}\,\cos \,{\phi }_{{\rm{M}}}+{\bf{t}}\,\sin \,{\phi }_{{\rm{M}}},\end{array}$$

yielding finally

16 $${{\bf{b}}}_{{\rm{Raman}}}\doteq ({s}_{1}\,\cos \,{\delta }_{{\rm{m}}}\,\cos \,{\phi }_{{\rm{m}}},{s}_{2}\,\cos \,{\delta }_{{\rm{M}}}\,\cos \,{\phi }_{{\rm{M}}},{b}_{3}),$$

where the unknown signs ${s}_{i}=\pm \,1$ should be chosen in a way that *B*1 and *B*2 axes are mutually orthogonal, which means that

17 $${s}_{1}{s}_{2}{\bf{e}}({\phi }_{{\rm{m}}})\cdot {\bf{e}}({\phi }_{{\rm{M}}})+{n}_{1}{n}_{2}=0,$$

what together with ${n}_{1} > 0$ and ${n}_{2} > 0$ implies that one should select ${s}_{i}=\pm \,1$ in agreement with

18 $${s}_{1}{s}_{2}{\bf{e}}({\phi }_{{\rm{m}}})\cdot {\bf{e}}({\phi }_{{\rm{M}}}) < 0$$

Finally, *b*_3_ can be determined from the orthonormality condition ${{\bf{b}}}_{{\rm{Raman}}}\cdot {{\bf{n}}}_{{\rm{Raman}}}=0$.

The obtained values of *λ*_M_ and *λ*_m_ for several selected grains from Fig. 3, together with the resulting local coordinates of vectors **n**, determined from equation (14), are given in Table 1. Additional information about the vectors **b**, obtained from ${\phi }_{{\rm{M}}}$ and ${\phi }_{{\rm{m}}}$ with the help of equation (16), are given in Table 2.

**Table 1:** Orientation of the grain with respect to the surface normal.

Grain	*λ*_M_	*λ*_m_	n_Raman_	n_EBSD_
A	0.98	0.99	0.07, 0.09, 0.99	0.06, 0.06, 1.00
B	0.87	0.24	0.58, 0.21, 0.78	0.66, 0.01, 0.75
C	0.92	0.50	0.44, 0.16, 0.88	0.49, 0.21, 0.85
D	0.58	0.27	0.57, 0.40, 0.72	0.59, 0.41, 0.69

Grains labels are those of Fig. 3, *λ*_M_ and *λ*_M_ are the reduced polarization factors in equation (11) at the principal and secondary maxima of the angular dependence of the intensity of the strongest *E*-symmetry Raman mode in the parallel-polarised Raman spectrum. The crystallographic coordinates of the surface normal unit vector **n**_Raman_ and **n**_EBSD_ are determined from Raman scattering and EBSD, respectively.

**Table 2:** Orientation of the grain with respect to the reference direction on the surface.

Grain	*φ*_M_	*φ*_m_	b_Raman_	b_EBSD_
A	27	−60	−0.50, −0.89, 0.12	−0.45, −0.89, 0.08
B	−41	48	−0.54, −0.74, 0.60	−0.52, −0.72, 0.46
C	−7	88	0.03, −0.98, 0.16	0.00, −0.97, 0.24
D	89	−10	0.82, 0.02, −0.66	0.74, 0.05, −0.67

Grains labels are those of Fig. 3, *φ*_M_ and *φ*_m_ are the angles (in degrees) of the principal and secondary maxima of the angular dependence of the intensity of the strongest *E*-symmetry Raman mode in the parallel-polarised Raman spectrum. The crystallographic coordinates of the reference direction vector **b**_Raman_ and **b**_EBSD_ are determined from Raman scattering and EBSD, respectively.

## Discussion

The grain orientations obtained from Raman scattering are in a quite good agreement with the results obtained from EBSD technique. It is worth noting that GaV_4_S_8_ is a semiconductor with a sizeable absorption at the wavelength of the laser used for the present Raman scattering experiment. Consequently, the Raman scattering is collected from the top submicron layer of the investigated surface. In more transparent materials, optic method may show more deviations from the EBSD technique simply because the Raman scattering would be averaging a deeper volume. To some extent, if needed, the penetration depth could be tuned by selection of the laser wavelength. The vertical resolution would be more coarse but, in principle, one can possibly seek information about the depth profile by using confocal Raman microscopy.

On the practical side, application of this Raman spectroscopy method generally requires an optically polished or a naturally flat incident surface and, in absorbing materials like GaV_4_S_8_, one have to limit the laser power in the focused area in a way to avoid local heating or even laser-induced surface degradation. Since cubic materials are optically isotropic, one might also expect that when using a uniformly polished surface of the pellet and when paying an attention to maintain the same focus, power and similar geometrical conditions, one could perhaps directly use the recorded *E*-mode intensities, without their normalisation to the intensity of *A*_1_-mode. However, in the case of our GaV_4_S_8_ ceramics, this strategy was not successful, and in spite of our utmost care, the overall scattering intensity considerably varied from grain to grain, and we had to use the relative mode intensities.

It should be noted that the protocol described above fails for grains accidentally oriented with domain normal very close to the $\langle 111\rangle$ directions, because then the $I(\phi )$ is constant and therefore the positions of the maxima cannot be well determined. However, the expression for the surface normal **n** is still reasonably estimated by this method. For determination of the in-plane orientation of such grain, that is for the **b**-vector coordinates, this protocol is not suitable, but one can still use the angular dependence of *F*-symmetry modes instead. In either case, if a high precision in crystal grain orientation is needed, one can always abandon equation (12) and resort to a direct numerical fitting of the measured $I(\phi )$ profiles to the exact expressions given by equations (5).

Finally, we would like to stress that the form of the Raman tensor given in equation (2) is common to a doubly degenerate Raman mode in any of the five cubic point groups. Therefore, the present method and the equations (7–16) are valid for all cubic crystals having a first-order Raman active mode transforming as a two-dimensional irreducible representation (as a mode of *E* or *E*_*g*_ symmetry). In particular, it applies to both centrosymmetric and noncentrosymmetric cubic crystal structures. Obviously, the simplest cubic structures like rock-salt or cubic perovskite have no Raman active modes at all. On the other hand, a similar approach can be adopted even for certain noncubic crystals, for example the angular dependence of the pure transverse optic mode of rhombohedral BiFeO_3_, located at 520 cm^−1^, seems to have almost identical polarization dependence with respect to the pseudocubic axes as the cubic doublet modes investigated here^16^. Nevertheless, as mentioned in the introduction, there are other, more straightforward optic and Raman scattering methods available for noncubic crystals, too.

In conclusion, we have realised that the strong polarisation dependence of Raman scattering by *E* or *E*_*g*_ symmetry modes in cubic materials could be used to determine crystallographic orientation of an arbitrarily oriented crystal facet. We have derived and tested approximate analytic expressions that allow of determining both the crystallographic orientation of the surface normal as well as the crystallographic orientation of a selected direction in the crystal surface. The method can be applied to any cubic material with *E* or *E*_*g*_ symmetry Raman modes. Comparison of Raman scattering and EBSD techniques applied to the same grains on a selected surface of GVS ceramics indicates that the method is functional and reasonably precise. The method can be very useful when a polycrystalline sample is used to determine anisotropic properties. In particular, we believe that this fully optical method can have advantage as a relatively cheap alternative to EBSD or in a limited experimental environment, for example when in-situ EBSD is not available but optical microscopy can be arranged.

## Methods

GaV_4_S_8_ was synthesised by direct single-step synthesis from elements. Sulphur and vanadium powders were mixed with lumps of gallium. The mixture was heated slightly by infrared lamp to melt gallium and thereafter ground and mixed in an agate mortar. The reaction mixture was put into the silica glass ampoule previously filled with argon. The ampoule was evacuated by a rotary pump, filled with argon, again evacuated, filled with argon, finally evacuated to residual argon pressure of about 2 mbar and sealed. The ampoule with reaction mixture was slowly heated at a rate of 40 K/h to 1270 K in a chamber furnace, annealed for 7 days and cooled at 10 K/min. The slow heating is necessary to allow the reaction of sulphur, since at quick heating the high pressure of sulphur can burst the ampoule. Unlike the experience of other authors this single-step synthesis resulted in pure single-phase GaV_4_S_8_ powders as was proved by X-ray diffraction. The powder was axially pressed at a pressure of 380 MPa. The sintering was done again in a sealed silica glass ampoule by the same annealing procedure as for the synthesis of powders. The resulting ceramic GaV_4_S_8_ pellet has 99% purity according to X-ray diffraction. Samples were cut from the sintered pellet with a diamond cut-off wheel and then mechanically polished using diamond suspensions with particle sizes of 9 and 3 *μ*m. Final mechanical-chemical polishing was performed using a solution of colloidal silica (Struers OP-S).

The electron microscopy imaging and EBSD measurements were performed at 15 kV using an FEI Quanta 3D FEG scanning electron microscope equipped with TSL/EDAX Hikari camera. The EBSD analysis was done using the GaV_4_S_8_ room temperature crystal structure data^17^.

Raman scattering measurements were carried out using a Renishaw Raman microscope with 514 nm Argon laser in back-scattering geometry and a very similar set-up as the one used in our previous systematic polarised Raman scattering investigations^18,19^.

## Data Availability

The datasets generated and/or analysed during the current study are available from the corresponding author on reasonable request.
